# Action of Artificial Digestive Fluids on Hæmoglobin

**Published:** 1904-04-16

**Authors:** 


### ACTION OF ARTIFICIAL DIGESTIVE FLUIDS ON HÆMOGLOBIN.

Professor Halliburton [1] publishes some experiments which he has carried out with a view to investigating the manner in which hæmoglobin is absorbed when given by the mouth. He prepared a large quantity of crystalline hæmoglobin from dogs' blood and divided it into several portions :

1. On keeping it at body temperature for some days it underwent no change.

2. On adding saliva to it and keeping the mixture at 40° C., there was no change in 24 hours, except that the crystals underwent solution.

3. On adding to a solution of the crystals an equal volume of 0·4 per cent. hydrochloric acid the solution showed almost immediately, and in the cold, the spectroscopic appearances of acid hæmatin. On keeping it at a temperature of 40° C. a swollen amorphous precipitate of acid hæmatin gradually separated out.

4. Pepsin solution was added to the mixture with hydrochloric acid, and the resulting appearances were just the same. On keeping the mixture at 40° C. the acid hæmatin gradually separated out as a precipitate, the appearance of which closely resembled that of "coffee grounds." In this case the digest within a few hours gave evidence of the presence of albumose and peptone derived from the hydrolysis of the proteid component of the hæmoglobin. These did not appear in the previous experiment where hydrochloric acid alone was added ; the dissolved proteid in that case was simply acid albuminate. The acid hæmatin which separated out in both cases as a precipitate is insoluble and therefore not very likely to be available for absorption.

5. Sodium carbonate was next added to another portion of hæmoglobin solution until 1 per cent. was present. To half of this a solution of trypsin was added ; the result in both cases was the same so far as the iron-containing portion of the pigment was concerned. In the specimen where trypsin was added there was naturally in addition the formation of proteolytic products. No immediate change in the pigment was produced, but on keeping at 40° C. the fluid became reddish-brown, and showed the absorption spectrum of alkaline hæmatin. Thus pancreatic digestion of hæmoglobin, *in vitro*, yields a substance which remains in solution and is presumably in a form better adapted for absorption. The same result was obtained when the brown residue obtained by the action of gastric juice on hæmoglobin was employed. The alkaline hæmatin thus formed by the action of pancreatic juice is readily diffusible through animal membranes, and is therefore in a suitable condition for rapid absorption. A further series of experiments was performed on rats. Control rats were killed before the experiments were commenced, others were fed for three weeks on bread and milk, and others again for a similar period on bread, milk, and hæmoglobin. The blood was then examined quantitatively for hæmoglobin and the red corpuscles were enumerated. In the control rats the average percentage of hæmoglobin and red corpuscles (taking normal human blood as 100) were respectively 85 and 52·2. In the rats fed on bread and milk the average percentages were 85·6 and 49·3. While in those fed upon bread, milk, and hæmoglobin, the average percentages were 98·8 and 70.

The bearing of these experiments upon the administration of hæmoglobin or its derivatives to anæmic patients is obvious.

[1] Brit. Med. Jour., April 9.

